# Inhibitory Effect of Astaxanthin on Oxidative Stress-Induced Mitochondrial Dysfunction-A Mini-Review

**DOI:** 10.3390/nu10091137

**Published:** 2018-08-21

**Authors:** Suhn Hyung Kim, Hyeyoung Kim

**Affiliations:** Department of Food and Nutrition, Brain Korea 21 PLUS Project, College of Human Ecology, Yonsei University, Seoul 03722, Korea; cigdoli2@naver.com

**Keywords:** astaxanthin, oxidative stress, mitochondrial dysfunction, disease prevention

## Abstract

Oxidative stress is a major contributor to the pathogenesis of various human diseases as well as to the aging process. Mitochondria, as the center of cellular metabolism and major regulators of redox balance, play a critical role in disease development and progression. Mitochondrial dysfunction involving structural and metabolic impairment is prominent in oxidative stress-related diseases. Increased oxidative stress can damage mitochondria, and subsequent mitochondrial dysfunction generates excesses of mitochondrial reactive oxygen species that cause cellular damage. Mitochondrial dysfunction also activates the mitochondrial apoptotic pathway, resulting in cellular death. Astaxanthin, a red-colored xanthophyll carotenoid, exerts an anti-oxidative and anti-inflammatory effect on various cell lines. In this manner astaxanthin maintains mitochondrial integrity under various pathological conditions. In this review, the inhibitory effects of astaxanthin on oxidative stress-induced mitochondrial dysfunction and related disease development are discussed.

## 1. Introduction

Nutritional oxidative stress can be induced by an imbalance between antioxidant defense and pro-oxidant load caused by an inadequate or excess nutrient supply that results from poor diet or bad eating habits [1]. Infection, inflammation, and tobacco smoking also induce oxidative stress in various tissues and cells [2,3,4,5]. Oxidative stress is a leading cause of various human conditions such as metabolic syndromes, and neurodegenerative, cardiovascular, inflammatory diseases, as well as age-related diseases. Mitochondrial dysfunction may be involved in the development of oxidative stress-associated diseases [6,7]. Maintaining structural and functional integrity of the mitochondria is essential for normal cellular function, because mitochondria play a key role in energy metabolism, as well as in maintaining cellular redox state and regulating apoptosis. Owing to the fact that mitochondria are the major source of reactive oxygen species (ROS), their dysfunction causes oxidative stress that drives the cells into a diseased state. Impaired mitochondrial function has been implicated in various human diseases including fatty liver disease [8,9], atherosclerosis [10], neurodegenerative diseases including Alzheimer’s and Parkinson’s [11,12,13], and inflammatory diseases [14]. Particularly, impaired mitochondria are regarded as the main mediator of the aging process and subsequent age-related diseases [15].

Astaxanthin, which is a red-colored pigment that belongs to the xanthophyll subclass of carotenoids, has a strong antioxidant capacity and can scavenge singlet oxygen and free radicals, and thus prevent lipid peroxidation [16]. Owing to its antioxidant ability and cell signal modulating properties, astaxanthin exhibits a variety of beneficial biological activities and effects. These include protection against UV damage, anti-inflammatory and immunomodulatory activity, alleviation of metabolic syndrome, cardio protective effects, anti-diabetic activity, prevention of neuronal damage, anti-aging and anti-cancer activity, as well as inhibition of cell membrane peroxidation [17,18]. In general, astaxanthin can exert an inhibitory effect on the development of oxidative stress-associated diseases and mitochondrial dysfunction. Mitochondria, a source of reactive oxygen species (ROS), could be a target of ROS under pathologic conditions. Herein, we review the antioxidant effects of astaxanthin which have been uncovered through recent advances made in research focusing on oxidative stress-mediated mitochondrial dysfunction and its connection to disease development.

## 2. Oxidative Stress and Mitochondrial Dysfunction

Mitochondria are the center of cellular energy production and the major source of ROS. Mitochondria produce superoxide anions as byproducts of electron leakage from the mitochondrial respiratory chain complexes I and III. Both superoxide anion and its product, hydrogen peroxide, are considered mitochondrial ROS (mtROS) [19]. Under normal physiological conditions, mtROS are removed by a cellular antioxidant defense system comprised of superoxide dismutase (SOD), catalase, and glutathione peroxidase. However, under pathological conditions, mtROS are overproduced, and a phenomenon occurs that leads to accumulation of excess oxidant radicals that damage mitochondria and cells [20]. Mitochondrial membranes and mitochondrial DNA are particularly susceptible to oxidative damage because of their close proximity to the site of ROS production and because of individual predisposing factors such as the high polyunsaturated fatty acid content of the mitochondrial membrane and the lack of mitochondrial DNA histones. Uncontrolled overproduction of ROS can overwhelm the cellular antioxidant capacity and impair the mitochondria [21].

Though the sequential relationship between oxidative stress and mitochondrial dysfunction remains to be fully elucidated, it is proposed that increased oxidative stress causes mitochondrial dysfunction, and consequently, increases the levels of mtROS. Zorov et al. [22] proposed the term ‘ROS-induced ROS release’ to indicate the positive feedback action of ROS in causing their further production in mitochondria. This model, involving a continuous cycle of ROS production, is currently accepted as being part of the mechanism for the pathology of ROS-mediated diseases.

Mitochondrial dysfunction, caused by oxidative damage, is represented by morphological changes and functional losses of the mitochondria. Structural changes such as swelling and fragmentation of mitochondria are often observed, and mitochondrial fission increases under pathologic conditions. Disruption of the mitochondrial membrane potential (MMP) is major sign of mitochondrial dysfunction. Loss of the MMP results in a defective mitochondrial electron transport chain, decreased metabolic oxygen consumption, ATP depletion, and low energy metabolism. Oxidative stress to the mitochondria can induce permeabilization, triggering the mitochondrial apoptotic pathway. Specifically, opening of the mitochondrial permeability transition pore (mPTP) results in the release of cytochrome c from mitochondria to the cytoplasm, which in turn activates pro-apoptotic caspases. Mitochondrial dysfunction often follows altered calcium homeostasis and mutation of mitochondrial DNA [23].

## 3. Diseases Associated with Oxidative Stress and Mitochondrial Dysfunction

Oxidative stress-induced mitochondrial dysfunction is closely related to inflammatory responses associated with various diseases. Mitochondrial dysfunction underlies the endless cycle of oxidative stress and inflammation, in which increase in oxidative stress under inflammatory conditions results in mitochondrial dysfunction and dysfunctional mitochondria trigger amplified oxidative burst and propagate inflammation [24]. Through increased mtROS production and mitochondrial leakage, mitochondrial dysfunction can induce expression of pro-inflammatory cytokines, increase responsiveness of cells to inflammatory signaling, induce a damage-associated molecular pattern, and activate the inflammasome [25].

The associations between mitochondrial dysfunction and the aging process and neurodegenerative diseases have been well characterized. The mitochondrial theory of aging, extending from the free radical theory of aging, suggests that oxidative damage to mitochondrial components results in accumulation of mtROS and insufficient energy metabolism, which are the major causes of aging [26]. The neuronal system is particularly vulnerable to oxidative stress owing to its high lipid content and metabolic rate. Oxidative damage to the mitochondria and subsequent mitochondrial dysfunction are manifested in age-related neurodegenerative diseases such as Alzheimer’s disease. In addition to metabolic failure, damaged mitochondria lose their membrane potential, become permeable and release cytochrome c, thereby activating caspases that induce apoptosis of neuronal cells [27].

In case of atherosclerosis, mtROS are overproduced, and they activate inflammatory signaling and damage endothelial cells. Mitochondrial DNA mutation and impaired mitochondrial enzymes correlate with atherosclerosis. Supplementation with mitochondria-targeted antioxidants can prevent mitochondrial dysfunction and help maintain endothelial cell integrity [28], and thus, it can be beneficial in the treatment of metabolic diseases such as diabetes [29], alcohol-and obesity-induced fatty liver disease [9], and nonalcoholic fatty liver diseases [30].

## 4. Astaxanthin: Biochemistry and Bioactivities

**Figure 1:**
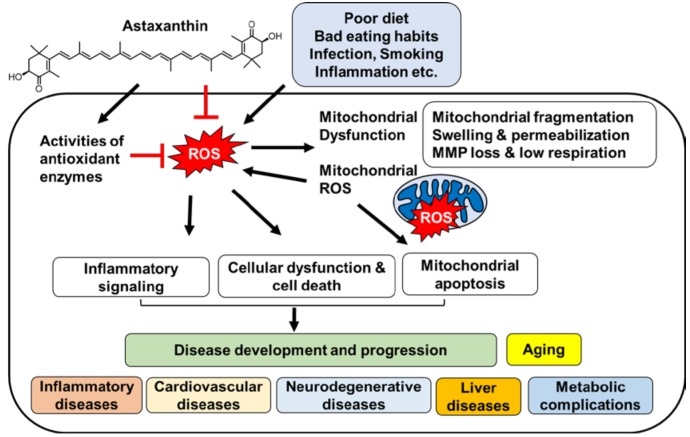
The proposed mechanism by which astaxanthin inhibits oxidative stress-induced mitochondrial dysfunction, and development and progression of diseases.

Astaxanthin is found in algae, yeast, and aquatic animals such as salmon, trout, shrimp, and lobster. The structure of astaxanthin (Figure 1) is comprised of a long nonpolar conjugated polyene backbone connecting polar ionone rings located at each end. As a result of its extended π-conjugation, astaxanthin is reactive towards reduction by free radicals and the presence of polar hydroxyl and carbonyl containing ionone rings gives it a higher antioxidant capacity than that of other carotenoids [18]. Furthermore, the polar-nonpolar-polar linear structural array enables astaxanthin to bind to and span the cell membrane [31].

Astaxanthin is usually derived from natural sources as an ester of fatty acids or as a conjugate of proteins in foods. Astaxanthin is absorbed into enterocytes through passive diffusion, and incorporated into chylomicrons to be delivered to the liver. This natural product is then incorporated into low-density lipoprotein and high-density lipoprotein and transported via circulation. Moreover, the bioavailability of astaxanthin is enhanced when taken together with dietary lipids [31,32].

Recent studies show that astaxanthin has a preventive effect of oxidative stress-induced degenerative pathological conditions. In a mouse model of Alzheimer’s disease, astaxanthin in the form of an ester with docosahexaenoic acid, reduces oxidative stress and inflammasome activation [33]. Astaxanthin decreases the oxidative stress level, as indicated by lowered plasma malondialdehyde levels, and reverses age-related changes in residual skin surface components of middle-aged subjects [34]. When it is administered as a supplement to type 2 diabetes mellitus patients, it improves the serum lipid profile, increases the adiponectin level, and decreases blood pressure [35]. Mortality and histological damage due to acute lung injury is improved by astaxanthin treatment and it decreases oxidative stress and the inflammatory response [36].

## 5. Effects of Astaxanthin on Oxidative Stress and Mitochondrial Dysfunction

Growing evidence suggests that astaxanthin can reduce oxidative stress and maintain mitochondrial integrity. Wolf et al. [37] proved that astaxanthin sustains mitochondrial function by protecting the mitochondrial redox balance. Astaxanthin significantly reduces physiologically occurring oxidative stress and maintains the mitochondria in a more reduced state, even after stimulation with H_2_O_2_. It also prevents loss of MMP and increases mitochondrial oxygen consumption. Astaxanthin might prevent mitochondrial dysfunction by permeating and co-localizing within mitochondria [38,39].

Astaxanthin reduces ROS levels and increases MMP in an in vitro model of inflammatory preeclampsia. Also, it prevents heat stress-induced impairment of blastocyst development by enhancing MMP [40]. In an in vivo study, oxidative damage was found to be mitigated, and compromised mitochondrial function is restored in geriatric dogs by astaxanthin treatment [41]. Astaxanthin treatment increases the mitochondrial content, ATP production, and respiratory chain complex activity, suggesting that it prevents aging by increasing mitochondrial efficiency [41].

Pongkan et al. [42] investigated the effect of astaxanthin on mitochondrial dysfunction in ischemic mice. The results showed that mitochondria isolated from ischemic myocardium of mice have higher levels of mtROS production and mitochondrial depolarization, and exhibit mitochondrial swelling. Treatment with astaxanthin reduces mtROS production, and mitochondria depolarization and swelling.

Astaxanthin was shown to inhibit cytochrome c release resulting from mitochondria permeabilization, and thereby, prevent mitochondria-mediated apoptotic death of cells. In a model of lung fibrosis, astaxanthin was observed to inhibit H_2_O_2_- and bleomycin-induced apoptosis of alveolar epithelial cells. Astaxanthin treatment protects mitochondrial inner and outer membranes, and the cristae against H_2_O_2_- or bleomycin-induced structural disruption, and improves MMP [43]. Astaxanthin also inhibits cytochrome c release and apoptosis of myocardial [44] and SH-SY5Y cells [45,46] by decreasing levels of ROS and consequent formation of protein oxidation products and by restoring MMP.

Lee et al. [47] investigated the neuroprotective effect of astaxanthin by using in vitro and in vivo models. These workers observed that the known neurotoxin, 1-methyl-4-phenylpyridinium (MPP+), induces neuronal cytotoxicity by causing oxidative stress-mediated opening of the mitochondrial permeability transition pore and subsequent release of cytochrome c. Astaxanthin suppresses MPP+-induced ROS generation by increasing SOD and catalase activities, thereby preventing a decrease in MMP and mPTP opening [47]. Wu et al. [48] showed that astaxanthin prevents opening of the mitochondrial permeability transition pore in SH-SY5Y cells by suppressing constitutive ROS production. Astaxanthin decreases ROS levels, particularly in the mitochondria, and prevents mitochondrial apoptosis by inhibiting cytochrome c release in total body irradiation injury [49] as well as in a burn-wound inflammation model [50,51].

## 6. Effect of Astaxanthin on Diseases Associated with Oxidative Stress and Mitochondrial Dysfunction

Oxidative stress, either as a consequence of increased production of ROS or depletion of the antioxidant enzyme system, leads to disease conditions. Often, mitochondrial dysfunction takes part in the onset of oxidative stress-associated disease. Astaxanthin is a powerful antioxidant, and as a result, it maintains metabolic efficiency of the mitochondria. Therefore, astaxanthin is a potential therapeutic agent for preventing or retarding disease progression.

Increased oxidative stress resulting from ROS/RNS and chronic inflammation are common features of cardiovascular diseases. In vitro- and in vivo-based studies showed that astaxanthin decreases ROS and RNS levels, decreases the formation of oxidative damage products, increases antioxidant enzyme activity, suppresses inflammatory signaling and reduces lipid peroxidation in the heart [52,53,54,55,56]. Astaxanthin significantly alleviates mitochondrial dysfunction associated with ischemic myocardial injury [42]. In particular, it restores mitochondrial integrity and inhibits mitochondria-mediated apoptosis in a homocysteine-induced cardiotoxicity model [44]. Homocysteine-induced overproduction of ROS, loss of MMP, and fragmentation of mitochondria are all prevented by astaxanthin pretreatment. Moreover, it normalizes the expression of Bcl-2 family proteins, thereby suppressing mediators of mitochondrial apoptosis such as poly-ADP-ribose polymerase and the caspases.

Neurodegenerative diseases are linked to oxidative stress and impaired mitochondrial efficiency. The constitutive cycle of ROS and free radical production causes substantial damage to the mitochondria, leading to mitochondrial failure. Antioxidants-rich diet can improve mitochondria redox status [57]. Because it can effectively cross the blood-brain-barrier, astaxanthin is an especially potent neural-protective agent in mammals [58,59]. Pretreatment of H_2_O_2_-stimulated mouse neural progenitor cells with astaxanthin inhibits apoptotic cell death and stimulates cell growth [60]. Astaxanthin leads to recovery of mitochondrial ATP production and blocks cytochrome c release by activating mitogen-activated protein kinase kinase (MEK) signaling and increasing the level of anti-apoptotic Bcl-2. Lu et al. [61] found that astaxanthin decreases the extent of ischemic infarction in rats by increasing the activities of antioxidants and by maintaining MMP.

Fat accumulation and oxidative stress impair the function of mitochondria via morphological alteration, increased membrane peroxidation, decreased ATP level, increased ROS production, defective mitochondrial β-oxidation and respiration, and increased mitochondrial permeabilization [62]. Based on its capacity to increase MMP and respiratory efficiency, astaxanthin is expected to rescue damaged mitochondria in nonalcoholic fatty liver disease (NAFLD). Astaxanthin reduces hepatic lipid accumulation and insulin resistance, and alleviates hepatic inflammation and fibrosis in nonalcoholic steatosis (NASH) mice [63]. Takahashi et al. [64] showed that astaxanthin accumulates in the liver, especially in the microsomal and mitochondrial fractions of the liver tissue. This substance has been shown to prevent oxidative damage to the liver, improve metabolic profiles, and reduce hepatic inflammation [65,66,67]. These studies suggest that astaxanthin might be an effective therapeutic for treatment of oxidative stress-mediated liver diseases.

Hyperglycemia and other metabolic syndromes that increase the risk of diabetes stimulate ROS production in mitochondria [68]. Oxidative stress leads to a chronic inflammatory state coupled with increased ROS production and these events cause cellular dysfunction and apoptosis in the pancreas, liver, endothelium and kidney [69,70]. Under oxidative stress conditions of hyperglycemia or insulin resistance, the mitochondrial ultrastructure is disturbed and its respiration is impaired [71,72]. Astaxanthin reduces hyperglycemia-induced ROS and RNS production, especially in the mitochondria [73]. Furthermore, it inhibits the inflammatory signaling and apoptosis occurring under diabetic conditions [74]. In human mesangial cells stimulated with high glucose levels, astaxanthin reduces mtROS production and inhibits high glucose-induced NF-κB activation. Astaxanthin also decreases the levels of 4-hydroxy-2,3-nonenal protein adducts, markers of lipids oxidation, in the mitochondria of human mesangial cells [75]. Studies have shown that astaxanthin alleviates diabetic complications such as neuropathy, retinopathy and nephropathy. Moreover, it reduces oxidative stress and inflammation and improves the metabolic profiles of glucose and fatty acids, thereby preventing cellular damage and dysfunction in the organs [76,77,78].

Taken together, these findings support the validity of the mechanisms summarized in Figure 1 for the inhibitory effect of astaxanthin on oxidative stress-induced mitochondrial dysfunction and disease development and progression. Certain stimuli such as poor diet, bad eating habits, infection, smoking, and inflammation, increase the levels of cellular ROS. Oxidative stress causes mitochondrial dysfunction associated with mitochondrial fragmentation, swelling and permeabilization, the loss of mitochondrial membrane potential, and decreased mitochondrial respiratory rate. Mitochondrial dysfunction leads to increase in mitochondrial ROS production, which in turn increases cellular ROS levels. Both cellular and mitochondrial ROS activate inflammatory signaling and induce cellular dysfunction and cell death. Increased levels of mitochondrial ROS cause mitochondrial apoptosis. Accordingly, oxidative stress is critical in the development and progression of inflammatory diseases, cardiovascular diseases, neurodegenerative diseases, liver diseases, and metabolic syndromes including diabetes as well as aging. Astaxanthin possesses ROS scavenging activity and it activates antioxidant enzymes. Consequently, it inhibits oxidative stress in cells caused by various stimuli. Therefore, astaxanthin suppresses oxidative stress-induced mitochondrial dysfunction, mitochondrial ROS production, and diseases development and progression. Table 1 shows the in vivo and in vitro studies on the effect of astaxanthin on oxidative stress-associated diseases and mitochondrial dysfunction.

## 7. Conclusions

Astaxanthin can effectively mitigate oxidative stress generated under various pathological conditions and prevent oxidative stress-induced mitochondrial dysfunction. Maintaining structural and functional integrity of the mitochondria can avert the onset and/or progression of human diseases. Astaxanthin can be considered as a potential therapeutic agent for treatment of pathological conditions associated with excess oxidative damage and dysfunctional mitochondria. Such conditions occur in cardiovascular diseases, neurodegenerative diseases, liver diseases including NAFLD, hyperglycemia and other metabolic syndromes including diabetes, diabetic complications such as neuropathy, retinopathy, and nephropathy, and inflammation in the pancreas, liver, endothelium and kidney. Finally, consumption of astaxanthin-rich foods might prevent metabolic complications related to the aging process.

## Figures and Tables

**Table 1:** In vitro and in vivo studies on the effect of astaxanthin on oxidative stress-associated diseases and mitochondrial dysfunction.

	Experimental Model	Effective Dose and Duration	Main Results	Reference
Inflammatory Diseases	Human umbilical vein endothelial cells (HUVECs) treated with H_2_O_2_	10 μM 48 h	cell viability ↑ reactive oxygen species (ROS) ↓ mitochondrial membrane potential (MMP) ↑	[40]
	Preeclamptic pregnant rats	25 mg/kg 16 days	blood pressure ↓ urinary protein ↓ oxidative stress marker; malondialdehyde (MDA) ↓ serum superoxide dismutase (SOD) ↑ histopathological changes ↓ preeclampsia-associated protein ↓ heme oxygenase-1 ↑ caspase-3 ↓ nuclear factor-κB (NF-κB) ↓	[40]
	Alveolar epithelial cells type II (AECs-II) from rats with bleomycin-induced lung fibrosis	1, 2 mg/kg 7 days	apoptosis ↓ SOD, catalase activities ↑ mitochondrial membrane integrity ↑ mitochondria swelling ↓ deformed cristae ↓	[43]
	Rat lung epithelial -T-antigen negative (RLE-6TN) cells treated with H_2_O_2_ or bleomycin	8 μM 6–24 h	apoptosis ↓ ROS ↓ SOD, catalase activities ↑ mitochondrial membrane integrity ↑ mitochondria swelling ↓ deformed cristae ↓ mitochondria disarrangement ↓ MMP ↑ pro-apoptotic protein ↓ anti-apoptotic protein ↑ cytochrome c release, caspase activation ↓ nuclear factor erythroid-derived 2-related factor 2 (Nrf2) ↑ p53 ↑	[43]
	A classic “comb” burn model in rats	5, 10, 20 mg/kg 48 h	burn-associated histological changes ↓ inflammatory cell infiltration ↓ oxidative stress marker (MDA) ↓ SOD, glutathione peroxidase ↑ xanthine oxidase, NADPH oxidase ↓ myeloperoxidase, TNF-α, IL-1β, IL-6 ↓ apoptosis ↓ activated cellular homolog of murine thymoma virus akt8 oncogene (Akt) ↑ inactivated Bcl-2-associated death promoter (Bad) protein ↑	[50]
	Severe burn rat model	5, 10, 20 mg/kg 24 h	histological and functional damage of kidney ↓ oxidation-reduction potential ↓ oxidative stress marker (MDA) ↓ SOD, catalase ↑ apoptosis ↓ activated Akt, inactivated Bad ↑ cytochrome c, caspases ↓	[51]
Aging	Geriatric dogs	20 mg/kg 16 weeks	oxidative stress markers (8-hydroxy-2′-deoxyguanosine, protein carbonyl, nitric oxide) ↓ blood SOD ↑ mitochondrial mass ↑ ATP production ↑ mitochondria Complex III production ↑	[41]
	Senescence accelerated mice (SAM)	8% of antioxidant diet 10 months	plasma glutathione (GSH) ↑ glutathione disulfide (GSSG) ↓ mitochondrial GSH in kidney, heart, brain, skeletal muscle ↑ ,mitochondrial GSSG in liver, kidney, heart, brain ↓ mitochondrial glutathione redox potential ↑	[57]
	Rats with d-galactose-induced brain aging	0.02% 8 weeks	oxidative stress markers (MDA, 8-hydroxy-2′-deoxyguanosine, protein carbonyls) in brain ↓ brain glutathione peroxidase, SOD activities ↑ total antioxidant capacity ↑ anti-apoptotic protein ↑ pro-apoptotic protein ↓ cyclooxygenase (COX)-2 ↓ brain-derived neurotrophic factor ↓	[59]
Cardiovascular Diseases	BALB/c mice	0.02, 0.08% 8 weeks	cardiac MMP ↑ TNF-α ↓ contractility of left ventricle ↑	[54]
	Human umbilical vein endothelial cells (HUVECs) exposed to glucose fluctuation	0.05, 0.1, 0.5 μM 3 days	ROS ↓ a component of NADPH oxidase p22^phox^ ↓ endogenous nitric oxide synthase (eNOS) ↑ nitrite ↓ peroxisome proliferator-activated receptor-γ coactivator (PGC)-1α ↑ IL-6, intercellular adhesion molecule-1 ↓ apoptosis ↓ phosphorylation of c-Jun N-terminal kinases (JNK), p-38 ↓	[55]
	Rats with isoproterenol hydrochloride-induced myocardial infarction	25 mg/kg 2 weeks	heart and kidney wet weight ↓ oxidative stress markers (MDA, nitric oxide) ↓ heart SOD, catalase, GSH ↑ histopathological changes ↓	[56]
	Mice with left anterior descending coronary artery (LAD) occlusion-induced ischemia-reperfusion injury	50 mg/kg 2 h	infarct size ↓ pro-apoptotic protein ↓ anti-apoptotic protein ↑ mitochondrial ROS ↓ cardiac mitochondria depolarization ↓ cardia mitochondria swelling ↓ oxidative stress marker (MDA) ↓	[42]
	H9c2 rat myocardial cells exposed to homocysteine	4 μM 6 h	cell viability ↑ apoptosis ↓ MMP ↑ mitochondria fragmentation ↓ pro-apoptotic protein ↓ anti-apoptotic protein ↑ intracellular ROS, mitochondrial ROS ↓ DNA damage ↓	[44]
	Homocysteine administered mice	5 mg/kg 4 weeks	GSH ↑ oxidative stress marker (MDA) ↓ apoptosis ↓	[44]
Neuro-degenerative Diseases	Human neuroblastoma SH-SY5Y cells treated with 6-hydroxydopamine	20 μM 30 min	apoptosis ↓ cytochrome c release, caspase-9 cleavage, caspase-3 activation ↓ p38 ↓ MMP ↑	[45]
	Human neuroblastoma SH-SY5Y cells treated with 6-hydroxydopamine or DHA hydroperoxide	100 nM 4 h	cell viability ↓ apoptosis ↓ cytochrome c release ↓ MMP ↑ oxidative stress marker (protein carbonyls) in mitochondrial fraction ↓ ROS ↓	[46]
	Human neuroblastoma SH-SY5Y cells treated with 1-methyl-4-phenylpyridinium (MPP+)	50 μM 25 h	cell viability ↑ apoptosis ↓ ROS ↓ SOD, catalase ↑ pro-apoptotic protein ↓ anti-apoptotic protein ↑ cytochrome c release, caspase activation ↓ MMP ↑	[47]
	1-Methyl-4-phenyl-1,2,3,6-tetrahydropyridine (MPTP)-induced mouse model of Parkinson’s disease	30 mg/kg 28 days	dopaminergic neurons ↑ histological hallmarks of Parkinson’s disease ↓	[47]
	Mouse neural progenitor cells treated with H_2_O_2_	10 ng/mL 8 h	apoptosis ↓ cell proliferation ↑ caspase activation ↓ ATP production ↑ mitochondrial leakage ↓ pro-apoptotic protein ↓ p38 ↑	[60]
	Primary cortical neuron treated with H_2_O_2_	500 nM 4 h	cell viability ↑ apoptosis ↓ MMP ↑	[61]
	Rats with middle cerebral artery occlusion (MCAO)-induced focal cerebral ischemia	50, 80 mg/kg 6 h	infarct volume ↓ neurological deficit score ↓	[61]
Liver Diseases	Nonalcoholic steatohepatitis (NASH) mice fed high-fat, cholesterol, and chocolate diet	0.02% 12 weeks	liver AST, ALT ↓ triglyceride, total cholesterol, non-esterified fatty acid ↓ hepatic lipid accumulation ↓ oxidative stress marker (MDA) ↓ lipogenic gene expression ↓ glucose intolerance ↓ hyperinsulinemia ↓ hepatic insulin signaling proteins ↓ JNK, p38, NF-κB ↓ infiltration and activation of Kupffer cells ↓ hepatic fibrosis ↓	[63]
	Rat model of ischemia-reperfusion injury	5 mg/kg 14 days	Histopathological score ↓ cell damage ↓ xanthine dehydrogenase: xanthine oxidase ratio ↑ mitochondrial swelling ↓ rough endoplasmic reticulum disarrangement ↓	[65]
	High fat- high fructose diet -induced mice obesity model	6 mg/kg 45 days	body weight ↓ hepatomegaly ↓ plasma glucose ↓ plasma liver lipid ↓ oxidative stress markers (MDA, nitrite nitrosothiol) ↓ SOD, catalase, glutathione peroxidase, glutathione s-transferase ↑ TGF-β1 ↓ histological abnormality ↓	[66]
	Rats intoxicated with CCL_4_	10 mg/kg 2 weeks	liver AST, ALT, alkaline phosphatase ↓ oxidative stress markers (MDA, nitric oxide) ↓ SOD, catalase activities ↑ myeloperoxidase ↓ inflammatory cell infiltration ↓ liver tissue necrosis ↓ hepatic fibrosis ↓	[67]
Metabolic Complications	Porcine proximal tubular epithelial cells (PTECs) exposed to high glucose	5, 10 μg/mL 24–48 h	cell viability ↑ cytotoxicity ↓ pro-apoptotic protein ↓ anti-apoptotic protein ↑ reactive nitrogen species (RNS) (•O_2_, NO•, ONOO–) ↓ oxidative stress marker (MDA) ↓ COX-2, inducible nitric oxide synthase (iNOS), NF-κB ↓	[73]
	Alloxan-induced diabetic rat model	20 mg/kg 30 days	blood glucose, blood triglyceride ↓ pro-reducing redox balance of plasmalymphocyte oxidative stress marker (MDA) ↓ lymphocyte ROS/RNS (H_2_O_2,_ •O_2_, NO•) ↓ calcium influx of lymphocytes ↓	[74]
	Normal human mesangial cells (NHMCs) treated with high glucose	10^−6^ M 24 h	mitochondrial ROS ↓ activator protein-1 activation ↓ monocyte chemoattractant peptide-1, COX-1, TGF-β1 ↓ lipid peroxidation in mitochondria ↓ mitochondrial protein adducts ↓ NF-κB ↓	[75]
	Streptozotocin-induced diabetic rats	10, 20, 40 mg/kg 5 days	body weight ↓ blood glucose ↓ oxidative stress marker (MDA) in cerebral cortex and hippocampus ↓ SOD, GSH ↑ eNOS, iNOS ↓ NF-κB, TNF-α, IL-1β, IL-6 ↓ caspase ↓ phosphoinositide 3-kinase/Akt ↑	[77]
